# Chromosome-scale genome of *Culicoides brevitarsis* provides a resource for comparative and vector biology studies

**DOI:** 10.1186/s13071-026-07436-8

**Published:** 2026-05-23

**Authors:** Khandaker Asif Ahmed, Melissa J. Klein, Leon Court, Rahul V. Rane, Tom K. Walsh, Stacey E. Lynch, Prasad N. Paradkar, Debbie Eagles, Gunjan Pandey

**Affiliations:** 1 CSIRO Australian Animal Health Laboratory (AAHL), Australian Centre for Disease Preparedness (ACDP), East Geelong, VIC 3220 Australia; 2 CSIRO Agriculture and Food, Black Mountain, ACT 2601 Australia; 3 CSIRO Health and Biosecurity (H&B), Australian Centre for Disease Preparedness (ACDP), East Geelong, VIC 3220 Australia; 4 CSIRO Environment, Black Mountain, ACT 2601 Australia; 5 CSIRO Health and Biosecurity (H&B), Parkville, VIC 3052 Australia

**Keywords:** *Culicoides brevitarsis*, Chromosome-scale genome, Genome assembly, Comparative genomics, Gene family evolution, Immunity, Arbovirus transmission

## Abstract

**Background:**

Biting midges of the genus *Culicoides* (Diptera: Ceratopogonidae) are key arbovirus vectors of human and animal health relevance. Understanding the biological and molecular factors influencing arbovirus transmission is essential for informing effective mitigation strategies. *Culicoides brevitarsis* Kieffer is of particular importance due to its close relationship to cattle, high population abundance in suitable sites, and demonstrated vector capacity for arboviruses. However, limited genomic resources have constrained investigation into its molecular and evolutionary biology, necessitating a chromosome-level genome assembly and comparative genomic analyses.

**Methods:**

A chromosome-level genome assembly of *C. brevitarsis* was generated using Oxford Nanopore long reads, polished with Illumina short reads, and scaffolded using Hi-C sequencing, followed by genome annotation, comparative dipteran genomics, immune gene characterisation, and evolutionary analyses of vector competence–associated genes.

**Results:**

A 129.5 Mb genome was assembled into three chromosomes with high contiguity (N50 = 43 Mb, L50 = 2) and high completeness (98% for Arthropoda BUSCO odb10 lineage). Genome annotation identified 11,708 genes, including 661 immunity-related genes, as well as genes associated with key functional categories such as metabolism, sensory perception, and host–pathogen interactions. Comparative gene family analyses across 12 dipteran genomes revealed strong family-level (Ceratopogonidae) and genus-level (*Culicoides*) evolutionary signatures. In *C. brevitarsis*, 405 expanded and 262 contracted gene families were enriched for functions associated with metabolism, regulation, locomotion, and stimulus response. Integration of transcriptomic datasets from *C. sonorensis* Wirth and Jones identified 82 orthologous genes in *C. brevitarsis* corresponding to genes previously associated with differential responses to bluetongue virus infection, including lineage-specific patterns of conservation and selection.

**Conclusions:**

The genome provides a foundational resource to understand the molecular biology and evolutionary features of *C. brevitarsis*. Integration of immune, sensory, metabolic, and evolutionary analysis provides a framework for future functional and comparative studies aimed at understanding variation in arbovirus transmission among *Culicoides* species.

**Graphical Abstract:**

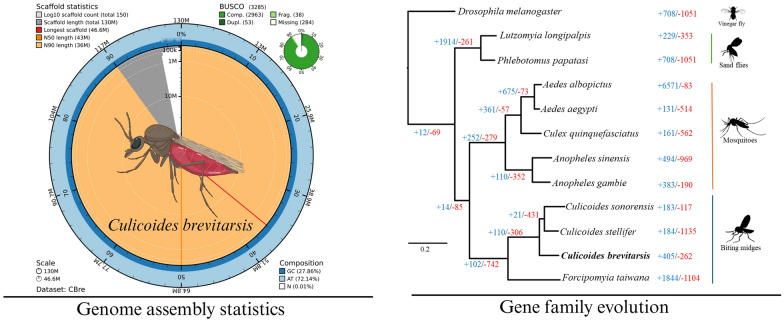

**Supplementary Information:**

The online version contains supplementary material available at 10.1186/s13071-026-07436-8.

## Background

Vector competence is a complex, multifactorial trait of arthropods, defined as the ability of a vector to acquire, support replication of, and transmit a pathogen to a susceptible host. Vector competence has been extensively studied in mosquitoes where intrinsic factors (e.g., host genetics, immune signaling, microbiome composition, and tissue barriers) interact with viral and environmental variables. [1]. In contrast, broader ecological factors such as host availability and environmental conditions contribute to vectorial capacity rather than vector competence. The genetic and molecular factors influencing vector competence remain comparatively less well understood in *Culicoides* spp. than mosquitoes, despite the global expansion of *Culicoides*-transmitted diseases [2]. Among these, bluetongue virus (BTV) is one of the most economically significant arboviruses affecting domestic ruminants, resulting in high mortality rates and substantial economic loss.

BTV is listed as a notifiable disease by the World Organization for Animal Health (WOAH), with annual global losses of more than 3 billion US dollars annually between 1960 and 2015 [3]. Epizootic and hyperendemic episodes of BTV have a global impact, with major outbreaks of disease recorded in Europe and India [2, 4–6]. In Australia, 13 out of 30 BTV serotypes have been detected [7–9], predominantly in the Northern Territory, Queensland, and the northern region of Western Australia. In Australasia, *C. brevitarsis* Kieffer is the most abundant and epidemiologically significant vector of BTV and is also implicated in the transmission of other arboviruses of veterinary importance. [10, 11]. Although its infection (0.3–1.5%) and transmission rates are poor [12, 13], its widespread distribution, high population density in livestock systems, and capacity for long-distance dispersal contribute to its central role in sustaining arbovirus transmission in Australia [14]. *C. brevitarsis* is thought to drive the periodic expansion of BTV-1 and BTV-21 along the east coast of Australia [15, 16].

In contrast to *C. brevitarsis*, the North American species *C. sonorensis* Wirth and Jones has been extensively studied due to the availability of laboratory colonies, which enable controlled investigations of virus–vector interactions [17]. These systems have provided key insights into infection barriers, immune responses, and gene expression associated with arbovirus transmission. In contrast, the absence of stable laboratory colonies for *C. brevitarsis* has limited experimental studies, restricting direct investigation of vector–virus interactions in this species.

Experimental studies have also demonstrated that vector competence in *Culicoides* is influenced by genetic factors, including maternal effect on BTV infection [18], as well as interactions between vector, virus, and environmental conditions [19–22]. However, these relationships are complex and cannot be attributed to single genes or pathways, reflecting a multifactorial phenotype shaped by both intrinsic and extrinsic factors. Despite its epidemiological relevance, research on *C. brevitarsis* has largely focused on arbovirus field surveillance, modeling of adult dispersal, vector competence assays, and host-feeding preferences. Early modeling studies demonstrated that environmental and climatic factors can strongly influence dispersal patterns and population structure, thereby modulating vectorial capacity [23, 24].

Experimental studies on the North American vector *C. sonorensis* have demonstrated that extrinsic factors such as temperature, viral strain, infectious dose, and environmental conditions strongly influence vectorial capacity. Intrinsic factors also play a critical role, including gut microbiota and the presence of a mesenteron (midgut) infection barrier, whereas a salivary gland barrier appears to be absent in *Culicoides* [25, 26]. In addition, heritable antiviral defenses, including immune signaling pathways, small interfering RNA (siRNA) responses, and melanization, contribute to vector competence for both BTV and epizootic hemorrhagic disease virus (EHDV) [2]. However, comparable insights into the genetic and genomic factors, including immune gene repertoires and their evolutionary dynamics, remain largely unresolved for *C. brevitarsis*, underscoring the need for high-quality genomic resources to investigate molecular and genomic features relevant to vector biology and host–pathogen interactions.

Recent advances highlight the value of molecular studies in *Culicoides* spp. Recently, Sharpe et al. [27] identified ten novel viruses in *C. brevitarsis* using RNA sequencing, providing tools for superinfection exclusion studies. Similarly, Ahmed et al. [28] reported mitogenomes of *C. brevitarsis*, *C. sonorensis*, and *C. imicola* revealing synteny in *C. brevitarsis* and *C. imicola*, compared with the distant North American species (*C. sonorensis* and *C. stellifer*). This phylogenetic study confirmed that Australian *C. brevitarsis* are phylogenetically close to *C. imicola* and may share biological and ecological similarities. Despite these advances, genomic resources remain heavily biased toward *C. sonorensis*. A study in 2018 [29] reported the first genome for this species, a fragmented scaffold-level assembly (189 Mb, Scaffold; *n* = 7974; N50 = 89 kb), which nonetheless enabled the identification of 165 genes previously associated with responses to BTV infection, including glutathione-S-transferase (*gst*) and the antiviral helicase (*ski2*). The genome has underpinned several studies in *Culicoides* spp., including the identification of differentially expressed genes under various feeding conditions [30] and the discovery of genes involved in the humoral immune response [31]. A more recent chromosome-level assembly (136.8 Mb, 189 Mb, Scaffold: *n* = 9; N50 = 45.8 Mb) has significantly improved continuity and completeness of the *C. sonorensis* genome (GenBank accession no. GCA_047716325.1). In parallel, another study [32] assembled the genome of a single *C. stellifer* individual, revealing a comparatively highly fragmented and smaller genome (119 Mb, Scaffold: *n* = 7974; N50 = 479.3 Kb) than that of *C. sonorensis*. However, no comparable high-quality genome exists for *C. brevitarsis* or other Australasian *Culicoides* spp., leaving a critical gap for comparative and functional genomics. Given its broad geographic distribution, morphological resemblance to the Afro-Eurasian vector *C. imicola*, and central role in the endemic transmission of BTV in Australia, a high-quality genome for *C. brevitarsis* is essential for investigating structural and gene-family evolution, and their roles in understanding genomic features relevant to vector biology and host–pathogen interactions.

In this study, the first chromosome-level genome assembly of *C. brevitarsis* from Australia is presented, generated using a combination of short-read (Illumina), long-read (Oxford Nanopore), and Hi-C sequencing technologies. Comparative genomic analyses across 12 dipteran taxa were conducted to investigate gene family evolution, functional enrichment of expanded and contracted gene families, and patterns of evolutionary divergence. This work provides a foundational genomic resource for *Culicoides* and supports future comparative and functional studies aimed at understanding biological factors associated with arbovirus transmission. It is important to note that this study does not experimentally assess vector competence in *C. brevitarsis*, and any associations with transmission-related traits are inferred from comparative genomic analyses.

## Methods

### Sample collection and DNA extraction

Samples for *C. brevitartis* used in this work were collected from Casino, New South Wales (latitude: −28.87, longitude: 153.05) using Polyvinyl Chloride (PVC) light traps (commonly known as Botraps) fitted with green ultraviolet (UV) lights, as routinely deployed in the National Arbovirus Monitoring Programme (NAMP). The collection protocol followed the standard collection protocol [33], except the collection bottles contained 100% ethanol, instead of the usual 80%, to preserve DNA integrity.

After overnight collection, the collection bottles were air-transported on dry ice to the high containment facility of Australian Centre for Disease Preparedness (ACDP), Geelong, Victoria (approximately 1670 km distant from the collection site) and the species was confirmed on the basis of morphology [34] and whole mitochondrial genome data as reported earlier [28]. A pool of 30 female *C. brevitarsis* was used for downstream molecular work. Females were selected as they are the blood-feeding sex responsible for pathogen transmission and are therefore of primary relevance for vector biology studies. In addition, field collections using light traps predominantly yield female specimens, facilitating sufficient sample acquisition for genomic analyses. Due to the small body size of *C. brevitarsis*, different sample preparations were required for Hi-C and sequencing libraries.

High molecular weight DNA was extracted using Qiagen Genomic Tip 20/G kit (Qiagen, USA), following user-developed “Isolation of genomic DNA from mosquitoes or other insects using the QIAGEN® Genomic-tip” protocol (available on Qiagen website), with additional washing with ethanol removal solution (400 mM NaCl, 20 mM Tris, pH 7.5, 30 mM EDTA; adjusted final pH to 8) to eliminate ethanol. DNA quality and quantity were measured using a Nanodrop (Thermo, USA) and Qubit dsDNA broad range quantitation assay kit (Cat no: Q32850; Invitrogen, USA).

### Genome sequencing

For Illumina short-read sequencing, Illumina PCR-based libraries were commercially constructed as per the manufacturer’s protocol at the Azenta-China (Suzhou facility) and sequenced to approximately 100-fold coverage (21 GB, 71 M raw reads) on an Illumina NovaSeq 6000 sequencer, S4 flow cell lane (2 × 150 bp PE).

For Oxford Nanopore Technology (ONT) long-read sequencing, a ligation-based genomic DNA library was prepared using a Ligation sequencing kit (Cat: SQK-LSK114, Oxford Nanopore, UK) and sequenced in a PromethIon flowcell (Cat: FLO-PRO114M, Oxford Nanopore, UK). The flowcell was run for 90 h. Basecalling was performed using Guppy v5.1 [35] under the dna_r9.4.1_450bps_sup.cfg model. The super-accurate basecalling with a minimum of 4200 bp read length and a minimum Q score of 10 threshold passed 104.22 GB data for downstream assembly. For HiC libraries, ten ethanol-preserved female *C. brevitarsis* were used. The library was prepared using Arima-HiC 2.0 kit (Arima Genomics, San Diego, USA) and sequenced in Illumina NextSeq sequencer (Illumina, Inc., San Diego, USA), P1 flow cell lane (2X150bp PE). Both ONT and HiC library preparations and sequencing were commercially sourced at the Biomolecular Resource Facility, Australian National University, Canberra, Australia.

### Raw data QC, genome assembly, and annotation

Adapter sequences were removed from the resulting reads using TrimGalore v0.6.6 [36]. The long-read sequences were trimmed using Porechop v0.2.4 [37]. Genome size was estimated from the k-mer distribution of the Illumina sequences with Jellyfish v2.3.0 [38] and GenomeScope v1.0.0 [39], using k-mers ranging from 15 to 25 bp, selecting the k-mer with the best model fit.

The long-reads were assembled using Raven [40] under default settings. The resulting genome assembly was polished with three rounds using long reads and three rounds using short reads with Racon v1.4.22 [41] under default settings. This was followed by six rounds of short-read-based polishing using Polca MaSURCA v4.0.7 [42] under default settings to obtain the final contig assembly. Chromosome-level scaffolding was performed as described previously [43]. The final contig assembly was scaffolded using ALLHiC v1 [44] on the basis of genomic topological information and manually curated with JuiceBox v1.9.8 . The assembly was checked for contaminants using NCBI Foreign Contamination Screen (FCS) toolkit (FCS-adaptor and FCS-GX; [45]), and any contaminating sequences were removed.

The *C. breviratis* genome was annotated by the NCBI RefSeq team using their Eukaryotic Genome Annotation Pipeline (EGAPx pipeline v10.2) [46]. This annotated genome and the annotation report are publicly available at the NCBI Datasets (annotation release: GCF_036172545.1-RS_2024_03). Publicly available *C. stellifer* and *C. sonorensis* unannotated genomes were annotated using NCBI’s EGAPx pipeline (egapx-alpha v0.3.2) with RNA seq data obtained from Sequence Reads Archives (ERR no.: ERR2171964, ERR2171966, ERR2171968, ERR2171970, ERR2171972, ERR2171974, ERR2171976, ERR2171978).

### Genome analysis

This assembly was assessed using BUSCO v5.2.2 [47] against the arthropoda, insecta, and diptera lineages’ gene sets. Repetitive sequence analysis was carried out using RepeatModeler2 [48] and RepeatMasker v4.1.2 [49]. Functional annotations of the longest proteins per genes were carried out using EggNOG-mapper [50, 51] with search criteria of a 0.001 minimum e-value, 60% identity, and minimum 50% query and subject coverage. The taxonomic scope was set to Diptera. Semantic similar Redundant Gene Ontology (GO) terms were removed from the output and offspring terms were summarized to higher level 2 Biological Process (BP), Molecular Function (MF) and Cellular Component (CC) terms with the GOSlim and GSEBase R packages [52]. The enrichGO function of the ClusterProfiler R package [53, 54] was used to identify the proteins in with different GO terms. Names and activities of proteins of interest were extracted from the enzyme-database website [55] (https://www.enzyme-database.org/class.php) and manually curated.

To identify immune-related genes in *C. brevitarsis*, a two-step approach was undertaken. First, genes annotated under the Gene Ontology term immune system process (GO:0002376) and the KEGG category Organismal Systems: Immune System were extracted from the functional annotation. Second, an orthology-based search was performed using immune-related genes from well-characterized insect species. Immunity and signaling gene groups from *Drosophila melanogaster* were manually curated from FlyBase [56], and the longest amino acid sequence per gene was retrieved (accessed 15 November 2025). In addition, for *Ae. aegypti, Ae. albopictus, Ar. subalbatus, and H. xiaojinensis*, the longest amino acid sequence per immune-related gene reported in previous studies [57, 58–61] was extracted and compiled into individual reference datasets. These six reference gene sets were used as queries in BLASTP searches against the *C. brevitarsis* proteome, applying an e-value threshold of 1e–10 to identify putative immune-related genes.

### Gene expansion and contraction analysis of orthologous gene families

For comparative analyses, the longest protein for each gene was identified, and the corresponding gene IDs and amino acid sequences were extracted to provide a single representative protein model per gene (ncbi_cds2nucfile.py script from Orthonome pipeline) [62]. Orthologous analysis was conducted using Orthofinder v2 [63] with default settings. Further, to estimate the number of expanded and contracted gene families, Orthogroups were filtered using the clade_and_size_filter.py script of Cafe5 programme [64]. The filter gene table and ultrametric tree file were used to generate the file report file in Cafe5. Phylogenetic trees with numbers of orthogroup gene families were generated using CafePlotter (https://github.com/moshi4/CafePlotter). Further expanded or contracted gene families for each species underwent functional annotation and subsequent GO enrichment analysis as mentioned in the previous section.

### Identification of vector competence-related genes and dN/dS estimation

To identify genes potentially associated with vector competence, publicly available transcriptomic data from a previous study [29] (BioProject: PRJEB19938) was used. Raw reads were quality-trimmed using TrimGalore [36] and assessed using FastQC [65]. A de novo transcriptome assembly was generated using the Trinity v2.13.2 pipeline [66], and transcript abundance was estimated in an alignment-free manner using Salmon [67]. Gene-level expression matrices were produced via the abundance_estimates_to_matrix.pl script within the Trinity suite. Differential gene expression analysis was conducted in the EdgeR R package [68, 69], incorporating low-expression, group-based filtering with filterByExpr, TMM normalization, and a GLM-based statistical model. A pairwise contrast comparing competent versus refractory samples was applied, and genes with |log2FC|> 3 and adjusted *P*-value < 0.05 were considered significantly differentially expressed. These unigenes were annotated via Basic Local Alignment Search Tool for nucleotide sequence (BLASTN) search against the annotated *C. sonorensis* genome and its CDS set.

For dN/dS analysis, a three-step custom pipeline (bash scripts; available from github—https://github.com/asifratul/culicoides_brevitarsis_manuscript_codes) was developed. The first script (Blast2CDSfasta.sh) performed blastp and reciprocal blastp searches using the longest CDS isoforms of annotated and competence-related *C. sonorensis* genes against nine dipteran proteomes, filtered 1:1 orthologs using awk, and extracted corresponding CDS using SeqKit [70]. The second script (DnDs.sh) translated the CDS to protein sequences, aligned them using MAFFT [71] and created codon-based alignments with Pal2Nal [72]. The third script executed PAML’s codeml [73] to calculate dN/dS ratios and associated standard errors across orthologs. Resulting values were compiled into a matrix and post-processed manually in R Studio v2025.09.2 and Microsoft Excel (Microsoft 365) for classification and visualization.

## Results

### Genome assembly statistics and completeness

For *C. brevitarsis*, multi-platform sequencing produced 140.38 Gb of ONT long reads (30.38 million reads), 71 million × 2 Illumina paired-end reads, and 24.5 Gb (~190 × coverage) of Hi-C data, enabling robust genome assembly and chromosome-level scaffolding.

**Fig. 1:**
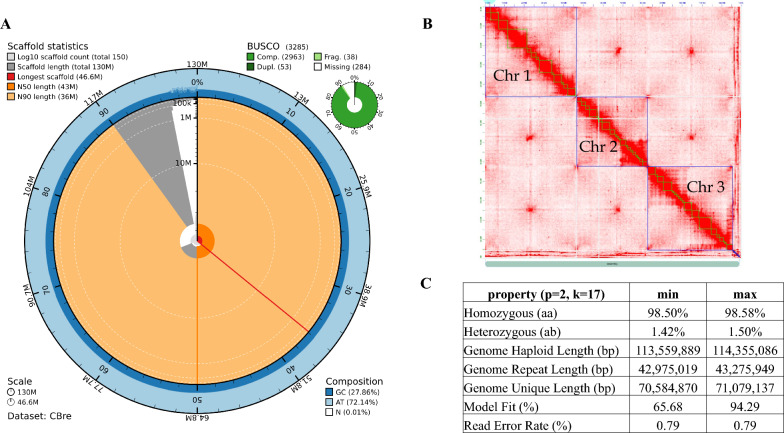
Assembly statistics and quality matrices of *C. brevitarsis* genome assembly. **A** A snail plot showing summary statistics and BUSCO scores, **B** HiC map, **C** Genome scope estimate for genome size estimation

Prior to genome assembly, the genome size and heterogeneity were estimated from 17-mer analysis of clean 150-bp paired end Illumina short read data. The genome size of *C. brevitarsis* was estimated at 113.6 Mb, with heterozygosity ranging from 1.41% to 1.50% (Fig. 1C). The primary genome assembly with PromethION long reads generated 129.6 Mb with N50 of 4.8 Mb and a Diptera BUSCO (odb_10) genome completeness of 88% (Fig. 1). Three rounds of polishing with long reads and six rounds of polishing with short reads resulted in the current assembly with 129.5 Mb length and largest fragment size of 46.6 Mb. The assembly was further improved using Hi-C data, which scaffolded 223 contigs (N50 = 3.5 Mb, L50 = 14) into 150 scaffolds (N50 = 43 Mb, L50 = 2), including three chromosomes. Overall, 99% of the nuclear genome sequences were found to be successfully anchored into three chromosomes and one mitogenome. We manually curated and annotated the mitochondrial genome from assembled contigs, which was estimated at 17.1 Kb in length [28].

Overall genome statistics and comparison with other *Culicoides* genomes are presented in Table 1. Three different genome assemblies of *C. sonorensis* are available from GenBank/RefSeq. The genome size varies, ranging from 155.9 Mb (GCA_900258525.3) to 209.4 Mb (GCA_900002565.1), which is significantly larger than *C. brevitarsis*. However, the genome size of the PacBio-based assembly of *Forcipomyia taiwana* is similar to the current assembly at 130.4 Mb. In terms of Benchmarking Universal Single-Copy Orthologs (BUSCO) completeness, the current assembly outperforms all other assemblies. The BUSCO analysis showed 98.1%, 97.1%, and 91.90% genome completeness for Arthropoda, insecta, and dipteran lineages (odb_10) [74], respectively (Table 1).

**Table 1:** Assembly and completeness statistics for *C. brevitarsis*, *C. stellifer*, *C. sonorensis*, and *F. taiwana* genomes

	*C. brevitarsis* (GCF_036172545.1)	*C. stellifer* (GCA_040583785.1)	*C. sonorensis* (GCA_047716325.1)	*F. taiwana* (GCA_963930915.1)
Assembly level	Chromosome	Contig	Chromosome	Scaffold
Sequencing technology	Illumina PE, ONT, HiC	PacBio Sequel	PacBio Sequel	Pacbio
Genome size (Mb)	129.5	119.3	136.8	130.4
Largest fragment (Mb)	46.6	1.73	48.1	7.58
Average fragment size (Mb)	0.9	0.27	13.68	1.15
N count	7421	0	700	22,736
Gaps (b)	86	0	4	30
Genome coverage (x)	80	99	40	42
Number of contigs	223	450	13	165
Contig N50 (Mb)	3.5	0.4793	32.6	2.5
Contig L50	14	81	2	17
Number of scaffolds	150	–	9	113
Scaffold N50 (Mb)	43	–	45	2.5
Scaffold L50	2	–	2	17
GC content (%)	28	31	28.5	43
Number of chromosomes	3	–	3	–
Chromosome 1 size (Mb)	46.6	–	48.1	–
Chromosome 2 size (Mb)	43	–	45.8	–
Chromosome 3 size (Mb)	36	–	41.7	–
Mitochondrion size (kb)	17.1	16.6	–	–
Arthropoda/Insecta/Diptera BUSCO (*n* = 1013/1367/3285)				
Overall score (S% + D%)	98.1/97.1/91.9	98.1/97.6/90	98.3/97.7/89.5	97.3/96.1/88.2
Complete and single-copy (S%)	96.2/95.7/89.3	95.8/95/87.9	97.2/96.5/88.4	75.8/74.4/68
Complete and duplicated (D%)	1.9/1.4/2.6	2.3/2.6/2.1	1.1/1.2/1.1	21.5/21.7/20.2
Fragmented (F%)	0.3/0.7/0.6	0.5/0.4/1.5	0.2/0.5/1.7	0.3/0.9/1.6
Missing (M%)	1.6/2.2/7.5	1.4/2/8.5	1.5/1.8/8.8	2.4/3/10.2

GCF accession numbers refer to NCBI RefSeq genome assembly identifiers corresponding to each species

### A summary of genome annotation

The RefSeq-based structural annotation of the *C. brevitarsis* genome predicted a total of 11,708 genes and pseudogenes, of which 11,137 (95.1%) and 508 (4.3%) were identified as protein- and noncoding genes, respectively (Table S1). Additionally, 63 (0.5%) nontranscribed pseudogenes and 1920 (16.4%) genes with known variants were also annotated. The transcriptome annotation yielded 15,549 transcripts, including 14,717 mRNAs (94.6%), 297 miscellaneous RNAs (1.9%), and various classes of noncoding RNAs: tRNA (207; 1.3%), lncRNA (241; 1.5%), snoRNA (13; 0.08%), snRNA (17; 0.11%), and rRNA (57; 0.37%). Exon and intron structures were well represented, with a total of 56,106 exons and 44,270 introns across the genome annotation. Coding transcripts accounted for the vast majority, with 54,974 exons (98.0%) and 43,476 introns (98.2%), whereas noncoding transcripts contributed 1883 exons (3.4%) and 1493 introns (3.4%). A total of 14,730 coding sequences (CDSs)/proteins were annotated, consistent with the high number of protein-coding genes. Gene structure metrics indicated a mean of 1.34 transcripts per gene (range: 1–40) and an average of 5.65 exons per transcript (range: 1–102), reflecting similar levels of transcript diversity and gene complexity compared with those reported for mosquito genomes, including *Aedes agypti* (1.8 transcripts per gene; 6.35 exons per transcript), *Aedes albopictus* (1.58; 5.53), *Culex quinquefasciatus* (1.59; 5.85), and *Anopheles gambiae* (2.31; 7.32), although with slightly lower transcript multiplicity.

### Repeated elements within the genome

A repetitive sequence analysis identified transposable elements and satellite repeats (Table 2). The repeated analysis identified 18.20 Mb (14.6%) of sequences as repeated, again similar to the *F. taiwana* genome (17.34 Mb, 13.30%) and *C. stellifer* (14.98 Mb, 12.56%), but almost half when compared with *C. sonorensis* assemblies (27.20 Mb, 19.88%), highlighting the unusual genome size shown in *C. sonorensis* assemblies. Among the transposable elements within current genome, most of them are retroelements (0.22%), which are further divided to Long Interspersed Nuclear Elements (LINEs) (0.2%) and Long Terminal Repeat (LTR) elements (0.02%) while DNA transposons occupied only 0.04% of the genome. A total of 12,324,169 bp (9.52%) interspersed repeats have been observed in *C. brevitarsis*. Simple repeats and low-complex sequences occupied 2.69% and 1.17% of the genome, respectively. A comparative analysis (Table 2) identified species-specific changes of retroelements and DNA transposons, where no SINEs were identified in *C. brevitarsis* and *F. taiwana* genomes, but a small amount in two of the three *C. sonorensis* assemblies.

**Table 2:** Repeat contents identified across *C. brevitarsis*, *C. sonorensis*, *C. stellifer*, and *F. taiwana* genomes

	*C. brevitarsis* (GCF_036172545.1)			*C. sonorensis* (GCA_047716325.1)			*C. stellifer* (GCA_040583785.1)			*F. taiwana* (GCA_963930915.1)		
Bases masked	18.20 Mb (14.06%)			27.20 Mb (19.88%)			14.98 Mb (12.56%)			17.34 Mb (13.30%)		
Elements	No.	Length (bp)	Percentage (%)	No.	Length (bp)	Percentage (%)	No.	Length (bp)	Percentage (%)	No.	Length (bp)	Percentage (%)
Retroelements	1569	289,910	0.22	11,605	3,399,224	2.48	635	282,824	0.24	2416	1,814,144	1.39
SINEs	–	–	–	–	–	–	–	–	–	–	–	–
Penelope	–	–	–	58	30,595	0.02	–	–	–	398	100,759	0.08
LINEs	1551	262,693	0.2	10,959	3,060,634	2.24	599	250,770	0.21	2025	1,338,142	1.03
L2/CR1/Rex	1021	166,728	0.13	3352	1,077,048	0.79	113	56,545	0.05	227	166,140	0.13
R1/LOA/Jockey	–	–	–	1538	414,257	0.3	–	–	–	134	126,302	0.1
R2/R4/NeSL	–	–	–	–	–	–	–	–	–	–	–	–
RTE/Bov-B	263	47,649	0.04	1742	419,747	0.31	170	22,558	0.02	322	451,944	0.35
L1/CIN4	–	–	–	48	15,737	0.01	–	–	–			
LTR elements	18	27,217	0.02	646	338,590	0.25	36	32,054	0.03	391	476,002	0.36
BEL/Pao	–	–	–	31	36,031	0.03	–	–	–	50	42,249	0.03
Ty1/Copia	–	–	–	108	113,156	0.08	36	32,054	0.03	44	16,769	0.01
Gypsy/DIRS1	18	27,217	0.02	507	189,403	0.14	–	–	–	246	371,108	0.28
Retroviral	–	–	–	–	–	–	–	–	–	51	45,876	0.04
DNA transposons	284	58,210	0.04	3250	664,726	0.49	1324	278,606	0.23	1752	517,632	0.4
hobo-Activator	–	–	–	2571	432,477	0.32	1324	278,606	0.23	1045	235,725	0.18
Tc1-IS630-Pogo	–	–	–	59	19,406	0.01	–	–	–	342	108,934	0.08
PiggyBac	–	–	–	83	38,932	0.03	–	–	–	–	–	–
Tourist/harbinger	141	27,039	0.02	–	–	0	–	–	–	191	111,974	0.09
Rolling-circles	1224	371,430	0.29	1550	419,650	0.31	899	174,521	0.15	2376	1,508,978	1.16
Unclassified	40,128	11,976,049	9.25	79,402	16,828,630	12.3	52,892	11,304,643	9.47	48,838	12,619,369	9.67
Total interspersed repeats	–	12,324,169	9.52	–	2,892,580	15.27	–	11,866,073	9.94	–	14,951,145	11.46
Small RNA	380	512,151	0.4	277	478,583	0.35	25	2951	0	125	144,451	0.11
Satellites	–	–	–	168	18,249	0.01	271	45,594	0.04	–	–	–
Simple repeats	76,918	3,480,198	2.69	96,994	4,058,905	2.97	53,459	2,275,363	1.91	15,138	597,343	0.46
Low complexity	27,741	1,515,330	1.17	27,103	1,336,567	0.98	10,821	615,996	0.52	2947	139,711	0.11

The table summarizes the proportion and classification of masked bases, including retroelements, DNA transposons, rolling-circle elements, simple repeats, and unclassified repeats. GCF accession numbers refer to NCBI RefSeq genome assembly identifiers corresponding to each species

### Functional inferences and enzymes

**Fig. 2:**
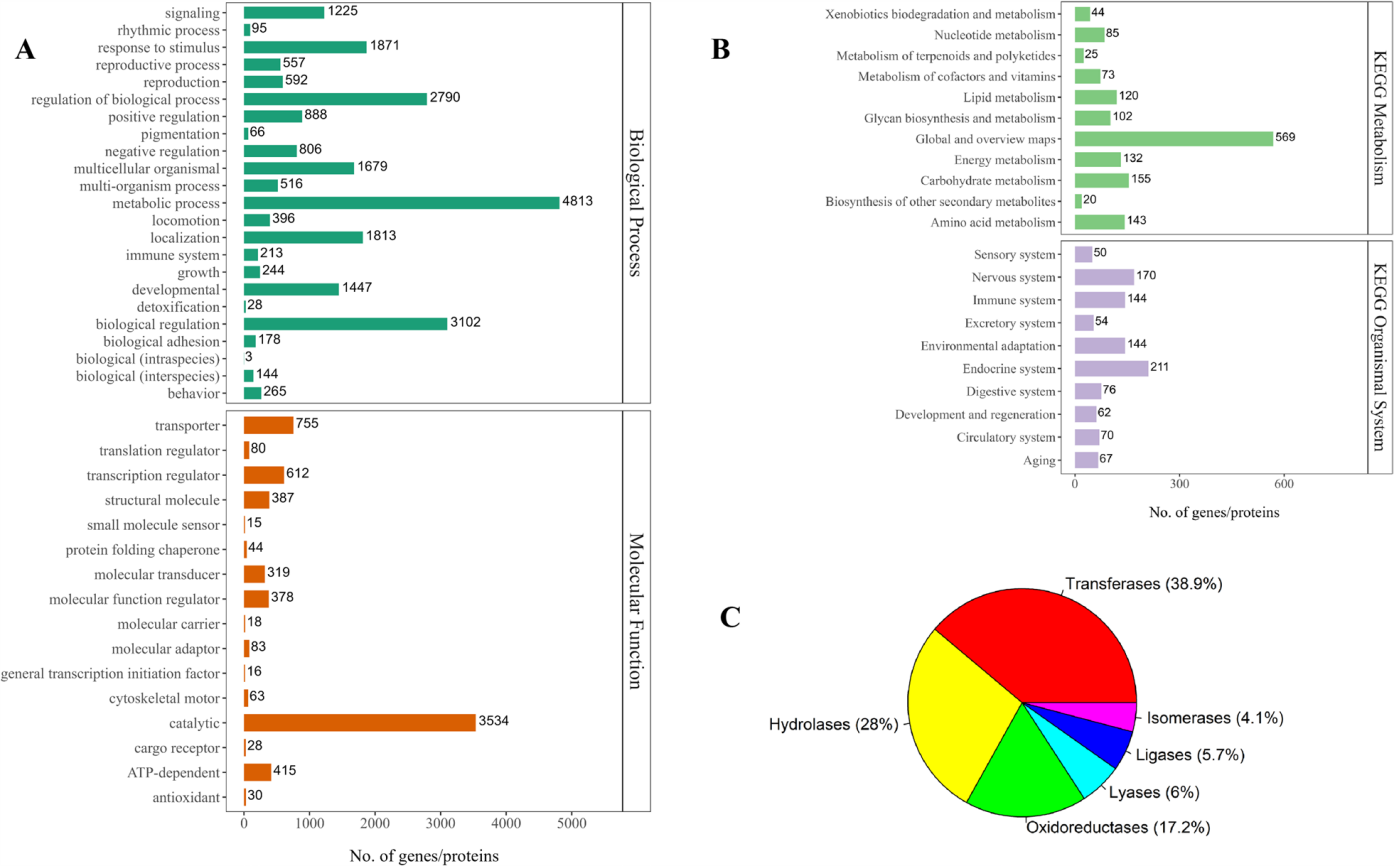
Functional annotation of *C. brevitarsis* proteome. **A** Biological processes and molecular functions **B** KEGG pathways, **C** Enzyme profile. The numbers are highlights number of genes/proteins under each gene ontology terms

For functional annotation, the longest protein per gene was retained as a representative sequence, consistent with previous genome, transcriptome, and proteome studies [75–78], resulting in functional inferences of 9471 proteins in the current genome. The functional annotation of the *C. brevitarsis* proteome revealed extensive coverage across biological processes, molecular functions, Kyoto Encyclopedia of Genes and Genomes (KEGG) pathways, and enzymatic classes (Fig. 2), highlighting the diverse physiological requirements of the species. In terms of biological processes (Fig. 2A), the most abundant proteins were associated with metabolic processes (4813 proteins; 17.1%), followed by biological regulation (3102; 11.0%) and regulation of biological processes (2790; 9.9%). Other prominent categories included response to stimulus (1871; 6.6%), locomotion (1813; 6.4%), and developmental processes (1447; 5.1%), which are likely to underpin key biological traits such as mobility, reproductive success, and host-seeking behavior. Notably, a smaller number of genes were linked to immune system processes (213 genes; 0.8%) and behavioral processes (265 genes; 0.9%), which may contribute to pathogen response, host-seeking, and feeding behavior in this species.

In the molecular function category (Fig. 2A), 3534 proteins (25.2%) displayed catalytic activity, underscoring the dominant role of enzymes in the proteome. Other prevalent functions included transporter activity (755; 5.4%), transcription regulation (612; 4.4%), and molecular transducer activity (319; 2.3%). However, 15 genes (0.1%) were annotated as small molecule sensors, potentially representing odorant-binding proteins or chemosensory receptors crucial for host-seeking. KEGG pathway mapping (Fig. 2B) assigned 1010 proteins (7.2%), related to global and overview metabolism, 249 and 166 proteins (1.8% and 1.2%, respectively) to carbohydrate and lipid metabolisms, reflecting a robust metabolic network supporting energy-demanding behaviors such as blood-feeding and flight. Within organismal systems, notable representation was seen in the endocrine system (489 genes; 3.5%), immune system (319; 2.3%), and nervous system (310; 2.2%), consistent with the neuroendocrine regulation and immune surveillance required in vector–host interactions.

A diverse enzyme profile was observed in the current genome, with all six major enzyme classes represented, reflecting the species’ broad metabolic versatility and ecological specialization (Fig. 2C). Transferases (38.9%) were the most abundant, mediating the transfer of functional groups such as methyl, glycosyl, or phosphate moieties. These enzymes, including glutathione S-transferases (GSTs) and UDP-glucuronosyltransferases, are pivotal for detoxification, insecticide resistance, and signal regulation. Hydrolases (28%), which catalyze the hydrolysis of various bonds, support nutrient digestion, cuticle degradation, and the breakdown of xenobiotics. Oxidoreductases (17.2%), such as cytochrome P450 monooxygenases, play central roles in metabolic resistance, hormonal synthesis, and oxidative stress management. The remaining enzyme classes, though less abundant, contribute important functions: lyases (6%) aid in intermediate metabolism and carbon processing; ligases (5.7%) are essential for DNA repair and biosynthesis; and isomerases (4.1%) enable structural rearrangement of biomolecules, crucial for metabolic homeostasis. Collectively, this enzymatic repertoire underscores the biochemical complexity of *C. brevitarsis* and is consistent with adaptive features associated with blood-feeding arthropods, including environmental resilience and potential for developing insecticide resistance.

### Identification of immunity-related genes

A total of 661 immunity-related genes were identified in *C. brevitarsis* through an integrated approach combining functional-ontology-based annotation and homology-based searches across multiple reference genera (Fig. S1, Table S2). The reference datasets comprised immune genes reported in *Drosophila melanogaster* (778 genes curated from FlyBase), *Aedes albopictus* (661 genes), *Aedes aegypti* (391 genes), *Armigeres subalbatus* (333 genes), and *Hepialus xiaojinensis* (258 genes), and were used exclusively as templates for immune gene identification. The highest recovery was obtained from orthology searches against *D. melanogaster* (365 genes; 46.9% of reported immune genes) and *Ae. aegypti* (177 genes; 45.3%). Intermediate recovery was observed for *Ae. albopictus* (165 genes; 25.0%), *Ar. subalbatus* (119 genes; 35.7%), and *H. xiaojinensis* (97 genes; 37.6%). In addition, functional-ontology-based annotation using GO and KEGG identified a total of 316 immune-associated genes. Largest overlaps were observed between *D. melanogaster* orthologs and GO/KEGG annotations (112 genes, 35.4% of the total 661 immune-related genes), followed by the overlap between *Ae. aegypti* and *Ae. albopictus* (35 genes, 5.3%). In contrast, only seven genes (1.1%) were conserved across all datasets (Fig. S1).

To further characterize the identified immunity-related genes, well-curated immune gene classifications of *D. melanogaster* were used as the primary reference framework. A total of 163 innate immunity genes were identified in *C. brevitarsis,* corresponding to 45.7% conservation relative to the innate immune gene set reported in *D. melanogaster* (Table 3). Core immune signaling pathways showed relatively high conservation, including Toll (51.3%), IMD (52.4%), and JAK–STAT (70.0%). Effector and execution mechanisms exhibited variabilities across categories, with strong conservation of superoxide dismutase (100%) and melanization-associated genes (70.2%), but markedly reduced conservation of antimicrobial peptides (4.0%). Genes involved in antiviral defense were among the most highly conserved categories, particularly RNAi-mediated antiviral responses (100%) and virus detection pathways (75.0%), highlighting the importance of antiviral immunity in *C. brevitarsis*.

**Table 3:** Immunity-related genes and their conservation between *D. melanogaster* and *C. brevitarsis*

	Reported in *D. melanogaster*	Ortholog detected in *C. brevitarsis*	Percentage conserved (%)
Innate immunity (overall)	357	163	45.7
Recognition and modulation			
Peptidoglycan recognition proteins (PGRPs)	13	3	23.1
Gram-negative bacteria binding proteins (GNBPs)	4	1	
C-type lectins	40	9	22.5
Nimrod	12	2	16.7
Serpin	17	6	35.3
cGAS-STING	6	4	66.7
Signaling pathway			
Toll	80	41	51.3
IMD	82	43	52.4
JAK-STAT	40	28	70.0
Effectors/execution			
Antimicrobial peptides (AMPs)	25	1	4.0
Lysozyme	17	4	23.5
Thioester-containing protein (TEP)	5	1	20.0
Peroxiredoxins	17	8	47.1
SOD	4	4	100.0
Melanization	57	40	70.2
Response to virus			
Virus detection	4	3	75.0
Cellular response	14	10	71.4
Defense response	65	36	55.4
Innate immune response	5	4	80.0
Regulation of response	20	14	70.0
RNAi-mediated antiviral response	5	5	100.0

The table summarizes innate immunity, recognition and modulation, signaling pathways, effector, and antiviral response gene categories reported in *D. melanogaster* and their corresponding orthologs identified in *C. brevitarsis*. The number of conserved genes and the percentage of conservation across functional classes are also shown

### Gene family expansion and contraction

**Fig. 3:**
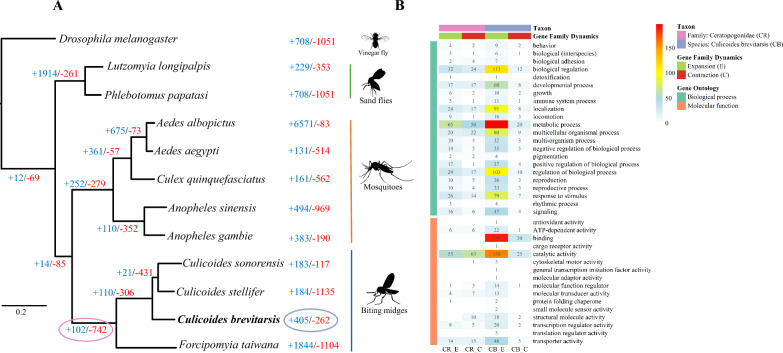
Gene family expansion and contraction across biting, non-biting, and mosquito genomes. **A** Phylogeny generated from single copy orthologues, identified across 12 proteomes. **B** Functional inferences of contracted (**C)** and expanded (**E)** proteins, identified across Ceratopogonidae family (CR) or *Culicoide brevitarsis* (CB) specific. Blue and red numbers represent number of expanded and contracted gene families

Expansion and contraction of gene families were identified across the analyzed dipteran species, providing comparative insights into lineage-specific genomic evolution and functional diversification. An orthology search was carried out across the overall proteomes of 12 dipteran species including mosquitoes (*Ae. aegypti*, *Ae. albopictus, Cu. quinquefasciatus, An. gambiae, An. sinensis*), biting midges (*C. brevitarsis, C. sonorensis, C. stellifer*), sand flies (*Lutzomyia longipalpis, Phlebotomus papatasi*), and the vinegar fly (*D. melanogaster*) as an outgroup. As genome annotations for *C. sonorensis* and *C. stellifer* are not publicly available at the National Center for Biotechnology Information (NCBI), de novo annotations were generated using an in-house workflow on the basis of the NCBI EGAPx pipeline. For *F. taiwana*, the annotation generated in the previous study was utilized [79]. The analysis revealed a total of 15,646 orthogroups, encompassing 159,546 proteins (92.4% of all predicted proteins). Only 7.6% of genes were unassigned. Of these orthogroups, 2427 (15.5%) were species-specific, involving 10,117 genes (5.9%), suggesting recent lineage-specific selection (Fig. 3A).

A core set of 4688 orthogroups was observed across all species, including 243 single-copy orthogroups, which were used for phylogenomic reconstruction. The three *Culicoides* species, along with the blood-feeding midge *F. taiwana*, clustered together as a distinct clade within the family Ceratopogonidae, forming a sister group to the Culicidae (mosquitoes). In contrast, sand flies (*L. longipalpis* and *P. papatasi*) belonging to the family Phlebotomidae were grouped separately within the suborder Nematocera. As expected, *D. melanogaster* served as an outgroup to all Nematoceran lineages. Across the Ceratopogonid lineage, a total of 102 gene families were expanded and 742 contracted, suggesting a trend of moderate innovation accompanied by substantial gene loss or streamlining. In comparison, the mosquito lineage (including Culicine and Anopheline) exhibited 252 expansions and 279 contractions, reflecting a more balanced pattern of genomic remodeling. Non-biting midges (Chironomidae) displayed the most dramatic gene family turnover, with 1914 expansions and 261 contractions, indicating extensive gene gain, possibly driven by adaptive radiation or environmental generalism.

Among the *Culicoides* genera, *C. brevitarsis* had the highest number of expanded gene families (+405) and moderate contractions (−262), followed by *C. stellifer* (+184/−1135) and *C. sonorensis* (+183/−117). The blood-feeding midge *F. taiwana*, another Ceratopogonid species, showed the highest number of expansions among midges (+1844) alongside substantial gene loss (−1104).

The predicted functional implications of gene family turnover within the family Ceratopogonidae, and specifically in *C. brevitarsis* (Fig. 3B), were further investigated. It is notable that members of this family occupy diverse ecological niches and have distinct evolutionary histories, with the Ceratopogonidae lineage dating back to at least the Early Cretaceous (~142 Ma) [80]; however, gene family-level comparisons provide a useful framework to identify broad functional trends and lineage-specific adaptations that may underlie ecological specialization. Across the Ceratopogonid lineage, the most notable changes were observed in gene families related to metabolic processes (65 expanded/50 contracted), biological regulation (32/24), response to stimulus (26/14), and localization (24/1). These patterns suggest active modulation of pathways involved in nutrient processing, environmental sensing, and intracellular transport. Functional shifts were also evident in developmental processes (17/17) and immune system processes (5/1), reflecting a balance between functional retention and reduction, likely tailored to the demands of growth, differentiation, and host–pathogen interaction. In terms of molecular functions, gene family turnover was particularly pronounced in catalytic activity (55/63) and transporter activity (14/15), indicating ongoing optimization of enzymatic function. Additional turnover in molecular transducer activity (4/7), structural molecules (0/10), and binding functions (22/30) suggests both functional refinement and loss of redundancy, consistent with ecological specialization within the group.

In the *C. brevitarsis* genome, gene family turnover showed a more pronounced and targeted pattern, compared with the other three Ceratopogonidae species included in this analysis. The most substantial changes were found in metabolic processes, with 197 gene families expanded and 20 contracted, highlighting enhanced metabolic flexibility to support energy production, blood-feeding, and reproductive functions. Likewise, biological regulation exhibited 113 expansions and 12 contractions in general functions, and 103/10 in regulatory subcategories, suggesting increased gene expression control coupled with selective simplification. Major changes also occurred in locomotion (91/3) and response to stimulus (79/7), functional domains essential for flight capacity, host-seeking, and environmental responsiveness. Immune-related functions (11/1) were moderately affected, reflecting improvements in immune surveillance and defense mechanisms. Gene families related to development (60/8) and multicellular organismal processes (80/9) also showed notable changes, associated with growth, differentiation, and transitions across developmental stages (e.g., larval, pupal, and adult). At the molecular level, substantial turnover was observed in catalytic activity (158/25), binding (189/30), and transporter activity (46/5), underscoring a fine-tuned biochemical landscape facilitating chemosensory functions, detoxification, and homeostasis.

### Evolution of competence-related genes

**Fig. 4:**
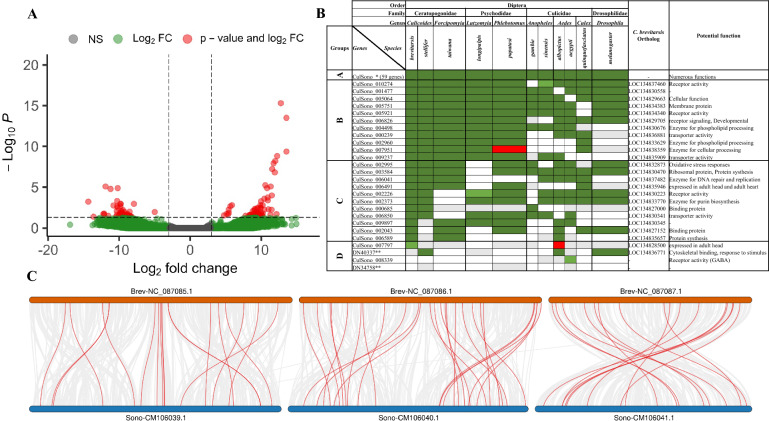
Evolutionary signatures of putative BTV vector-competence-associated genes across *Culicoides* and dipteran taxa. **A** Volcano plot showing differential expression patterns of BTV vector-competence candidate genes in *C. sonorensis*. **B** Presence and levels of divergence of vector competence-related genes across Dipteran taxa, with red cells indicating dN/dS > 1.00, grey indicating no significant deviation from neutrality, and green indicating dN/dS < 1.00 (light green: dN/dS > 0.40; dark green: dN/dS < 0.20; intermediate: 0.20–0.40). *C. brevitarsis* ortholog and potential functions are summarized on the right. **C** Synteny relationships of these vector competence-related genes between *C. brevitarsis* (Brev) *and C. sonorensis* (Sono) chromosomes

To identify genes associated with vector competence, the transcriptomic dataset of *C. sonorensis* reported by Ramiro et al. (2018) was used, which compared gene expression profiles between BTV competent and refractory females. In the original study, reads were aligned to a draft genome assembly, and differential gene expression analysis identified a total of 165 candidate genes. However, due to the unavailability of the original data files and the highly fragmented nature of the draft genome, the transcriptome was re-assembled using the Trinity pipeline to ensure consistency and improved contiguity. The re-analysis identified 141 differentially expressed unigenes, meeting the criteria of |log₂ fold change|> 3 and adjusted *P*-value < 0.05 (Fig. 4A). Of these, 86 unigenes were upregulated (mean log₂FC = 9.5; range 4.73–13.55) and 55 were downregulated (mean log₂FC = −9.75; range −4.98 to −14.34). Functional annotation of these unigenes against in-house *C. sonorensis* protein reference identified 84 corresponding protein models, while two unigenes remained unplaced. Interestingly, 55 unigenes did not contain identifiable open reading frames (ORFs). This observation may be attributed to biological factors, such as the presence of noncoding RNAs or pseudogenes or technical limitations, including assembly artifacts, low read coverage, or truncation during transcript reconstruction. Further investigation into these unannotated unigenes may provide insight into potential regulatory elements involved in vector competence.

To investigate the evolutionary dynamics of genes associated with vector competence, the dN/dS ratios were examined across orthologous proteins identified in ten dipteran species (Table S3). Overall, the majority of candidate genes exhibited a moderate-to-strong level of sequence conservation, with dN/dS ± SE < 0.019 in more than 95% of cases. Within the Ceratopogonidae family, 82 orthologous proteins were identified in *C. brevitarsis*, followed by 79 in *C. stellifer* and 77 in *F. taiwana*, reflecting the expected phylogeny-based conservation among closely related biting midges. Among non-biting midges (Psychodidae), 74 and 77 orthologs were detected in *L. longipalpis* and *P. papatasi*, respectively, indicating moderate conservation. Within the Culicidae lineage, *An. gambiae* exhibited the lowest number of orthologs (68), whereas *Ae. albopictus* retained the highest (76) among mosquito species. As expected, the most distant species in the analysis, *D. melanogaster,* showed low orthology, with only 71 orthologous proteins identified, consistent with its greater evolutionary divergence from the Ceratopogonid lineage. This suggests that genes potentially associated with vector competence are largely evolving under strong purifying selection, maintaining their functional integrity across divergent taxa.

To explore lineage-specific patterns, the orthologs were classified into four groups on the basis of taxonomic presence and selection pressure:

*Group A* consisted of 59 proteins conserved across all ten proteomes (Table S3, Fig. 4B), with consistently low (<0.019) dN/dS values. These genes were functionally enriched in enzymatic activity, protein binding, ribosomal structure, and transporter functions, suggesting they represent core molecular machinery required for cellular homeostasis and are likely to be operating under strong functional constraint, irrespective of vector status.

*Group B* included 11 proteins conserved across both midge families (Ceratopogonidae and Psychodidae) but showed divergence in mosquitoes (Culicidae) and/or fruitfly (*D. melanogaster*). Despite this divergence, these proteins mostly exhibited low dN/dS values (<0.019), suggesting stabilizing selection within midge lineages. Notable genes in this group included those encoding three enzymes with phospholipid processing and cellular processing, three proteins related to receptor and two transporter proteins. One protein kinase (CulSono_007951), with potential function of cellular processing, was conserved across both midge families but exhibited an elevated dN/dS in *P. papatasi*, potentially reflecting lineage-specific functional adaptation. Two proteins in this group remained uncharacterized.

*Group C* comprised 11 proteins with conservation largely restricted to the *Culicoides* genus, showing either absence or divergence in all other dipteran taxa. Despite this restricted distribution, dN/dS values remained low, suggesting these genes may be under purifying selection within *Culicoides*, potentially contributing to lineage-specific roles in vector competence. This group included two enzymes with roles in DNA repair/replication and purin biosynthesis, two binding proteins, and proteins with functions in protein synthesis, binding, and transportation. No functional interferences were found for CulSono_006491 and CulSono_009897, while the ortholog of CulSono_006491 in *D. melanogaster* showed high expression in the head and heart, which might indicate their tissue-specific role in both tissues. Nine occurrences of this group showed large SE values, which is why their calculations were deemed for this dN/dS analysis.

Finally, *Group D* included four uncharacterized proteins, two of which represent novel unigenes (DN40337 and DN34758) specific to *C. sonorensis*. These genes showed limited ortholog presence in other *Culicoides* species. CulSono_007797, an uncharacterized protein, showed orthologs only in two species, with a low dN/dS value in *C. brevitarsis* but a high dN/dS ratio (>1) in *Ae. albopictus*, suggesting possible positive selection or relaxed constraint in mosquitoes and functional conservation in *Culicoides*. This pattern may reflect a subgenus-specific adaptation associated with vector competence, potentially lost in other lineages. Together, these results highlight evolutionarily conserved and lineage-specific patterns in competence-associated genes across biting midges, providing a foundation for further investigation into their functional roles in arbovirus transmission.

## Conclusions

This study reports the first high quality, chromosome-level genome of *C. brevitarsis*. The total genome assembly size and overall GC content of *C. brevitarsis* are similar to those reported for *C. stellifer, C. sonorensis*, and *F. taiwana.* In terms of scaffold metrics, the *C. brevitarsis* genome showed a higher number of scaffolds, but conserving similar N50 and L50 metrics to *C. sonorensis*, which indicates, even though the scaffold number varied, the continuity of current genome is comparable to other high-quality assemblies. The current study identified three chromosomes, covering 99% of the genome. Three chromosomes were also evident in the physical map of the *C. sonorensis* (previously *C. variipennis*) genome, where probe-based markers unambiguously detected the presence of three distinct chromosomes [81, 82]. Chromosome-scale comparisons indicated broadly similar chromosomal organization between *C. brevitarsis* and *C. sonorensis*. However, *C. sonorensis* chromosomes appear slightly larger, consistent with its marginally greater genome size (136.8 versus 129.5 Mb). These metrics align with its highly contiguous assembly (contig N50: 32.6 Mb), compared with 3.5 Mb in *C. brevitarsis*.

In terms of BUSCO completeness, this genome outperformed all other openly available biting midge genomes. Compared with *C. stellifer* and *C. sonorensis* genomes, the *C. brevitarsis* genome has high complete/single copy BUSCO genes, and lower percentages of fragmented and missing genes indicating superior completeness. For duplicated BUSCOs, the genome showed similar metrics to *C. stellifer*, which are twice that of *C. sonorensis*. This might be due to differences in sequencing technologies or an underlying biological reality, which can be followed up in future studies. It is noteworthy that more than tenfold-duplicated BUSCO genes were evident in *F. taiwana*. Similarly high frequencies of duplicated BUSCO genes were observed in the earlier *C. sonorensis* genome, generated more than a decade ago using short-read sequencing of pooled samples [29]. Even though the assembly was generated from pooled samples, the assembly showed less heterogeneity, which reduced the number of duplicated genes significantly.

Repeat analysis identified a moderate number of repeated elements in *C. brevitarsis*, which is slightly higher than *C. stellifer* [32] and *F. taiwana* [79], but less than *C. sonorensis*. While major elements were co-detected across all the genomes, species-specific patterns are evident across different classes. Even though percentages of overall repeated elements varied, the number of unclassified repeats is quite similar to *C. stellifer* and *F. taiwana.* The highest repeated element is observed in *C. sonorensis*, with many unclassified and DNA transposons. While differences in sequencing technologies and sample pooling often vary the number of these elements, further study is required to investigate the root cause.

Previous annotations for *C. sonorensis*, *C. stellifer*, and *F. taiwana* were generated using MAKER and BRAKER3-based workflows, reporting 15,612, 18,895, and 16,898 protein-coding genes, respectively [29, 32, 79], whereas *C. bresitarsis* demonstrated 11,137 protein-coding genes. Castellanos-Labarcena et al. [32] further claimed that the higher number of predicted proteins in *C. stellifer* reflects superior assembly accuracy and increased reliability of BRAKER3 relative to the annotation of *C. brevitarsis*. However, this conclusion is confounded by methodological differences, as their comparison draws on BRAKER3-derived gene models for *C. stellifer* versus RefSeq/EGAPx-based annotation for *C. brevitarsis*, rendering the inference invalid. Gene counts are strongly pipeline-dependent; therefore, such claims cannot be interpreted as biological differences without harmonized annotation. To enable a fair comparison, both *C. stellifer* and *C. sonorensis* were reannotated using the same EGAPx workflow applied to *C. brevitarsis*. EGAPx can annotate 7758 and 11,266 proteins from *C. stellifer* and *C. sonorensis,* which indicates the completeness of the proteome, similar to these *Culicoides* spp. The absence of a *C. stellifer* transcriptome necessitated the use of the *C. sonorensis* transcriptome, resulting in fewer identified proteins. Additionally, the contig-level assembly of *C. stellifer* may have contributed to this discrepancy. Protein-coding gene counts in midge genomes are generally lower than in mosquito genomes, likely due to their larger genome size. Despite the reduced number of genes and proteins, GO analysis identified a broad range of proteins involved in key biological and molecular processes.

Functional inferences identified several immune-related, detoxification, and signaling proteins, important for vector biology and host–pathogen interaction of *C. brevitarsis.* Altered expression of immune-related genes often regulates susceptibility of arboviruses in mosquitoes and detoxification genes, e.g., P450, GST, and esterases often shape vectorial capacity of hosts [1, 83]. Signaling proteins impact salivary gland functions and virus dissemination across the body [84]. The analysis also detected several KEGG pathways, including the immune system and nervous system pathways. Immune proteins contribute to antiviral defense mechanisms, limiting virus transmission and survivability, while nervous system proteins linked to olfaction behaviors and host-seeking behavior, which is a major determinant for transmission efficacy and overall vectorial capacity [85–88]. Similar to other insect systems, current analysis identified several enzymes that modulate diverse functions. In the context of vector competence, transferases, hydrolases, and oxidoreductases are noteworthy enzymes. Transferases often affect virus entry and immune evasion, while hydrolases relate to pathogen–gut interaction by digestion. Oxidoreductases maintain redox balances, shaping pathogen survivability [89, 90].

A total of 661 immune-related genes have been identified in *C. brevitarsis*, exhibiting varying levels of conservation across different insect lineages. Notably, the non-Drosophila reference datasets were largely derived from earlier and now obsolete resources, such as the Insect Innate Immunity Database (IIID) [91], which focused on a limited subset of immune-related genes originally characterized in a small number of insect species. This historical bias likely contributes to the reduced overlap of immunity-related genes observed among the non-Drosophila datasets. In contrast, comparison with *D. melanogaster* identified 365 orthologous genes, including 163 innate immunity-related genes, highlighting substantial conservation despite deep evolutionary divergence. Recent advances in functional genomics, including in vivo gene knockdown and CRISPR–Cas9 approaches, have enabled detailed characterization of antiviral immunity in *D. melanogaster* and a limited number of mosquito species, revealing conserved roles for small RNA pathways, inducible immune signaling, autophagy, and transcriptional regulation. In *C. brevitarsis*, homologs of core antiviral components were identified, including JAK–STAT pathway genes (*Dome, Hop, STAT92E*), Toll pathway genes (*MyD88, Rel1, Cactus*), IMD pathway genes (*PGRP, DREdd, Caspar*), and autophagy-associated genes (*Toll-7, Atg6, Atg7, and Atg18*), which have been experimentally implicated in antiviral defense against a wide range of RNA and DNA viruses, including Sigma virus, Zika virus (ZIKV), Rift Valley fever virus (RVFV), Vesicular stomatitis virus (VSV), Sindbis virus (SINV), Drosophila X virus (DXV), and Drosophila C virus (DCV) in *D. melanogaster*, dengue virus (DENV) in *Ae. aegypti*, and O’nyong-nyong virus (ONNV) in *An. gambiae* [92]. These findings highlight genomic features associated with vector biology and host–pathogen interactions; however, their specific functional roles in arbovirus infection and transmission in *Culicoides* remain to be experimentally validated.

Phylogenetic analysis based on 243 single-copy orthogroups across 12 insect genomes showed clustering consistent with established taxonomic relationships, clearly separating Ceratopogonidae (*Culicoides* and *Forcipomyia*) from Culicidae (*Aedes, Culex*, and *Anopheles*), Drosophilidae (*Drosophila*), and Psychodidae (*Lutzomyia* and *Phlebotomus*). A similar phylogeny was also observed in *F. taiwana* [79], but limited to only six genomes. Within Ceratopogonidae, *C. sonorensis* and *C. stellifer* clustered more closely with each other than with *C. brevitarsis*, consistent with previous mitochondrial genome analyses [28]. Comparative analysis also identified gene family expansions and contractions across lineages, including in *C. brevitarsis*. These gene families were associated with general biological processes such as metabolism, regulation, and binding functions; however, their specific functional roles and evolutionary drivers remain to be further investigated.

The BTV competence-related transcriptome from *C. sonorensis* was reanalyzed to investigate the potential co-evolution of these genes across other *Culicoides* and insect genomes. Orthology-based searches identified > 95% of these *C. sonorensis* genes in the *C. brevitarsis* genome. Comparative synteny analysis of three chromosomes of *C. brevitarsis* and *C. sonorensis* revealed extensive collinearity, although multiple inversions and translocations were evident, particularly in chromosomes two and three of both genomes. While many of these genes were conserved across other mosquito and midge genomes, lineage-specific evolutionary patterns were also observed. dN/dS analysis indicated that most genes potentially involved in vector competence are under strong purifying selection (dN/dS < 0.19); however, on the basis of presence/absence patterns, four broad categories could still be distinguished. Among the analyzed insect genomes, 59 genes were universally present, 11 genes were conserved in Ceratopogonidae and Psychodidae families but absent in distant families (Culicidae and Drosophilidae), another 11 genes were conserved in Ceratopogonidae, and 4 found in *C. sonorensis*. While these gene sets have diverse functions, including receptors, transporters, or enzymes, these genes set will be instrumental to characterize the genetic basis of vector competence in biting insects and mechanisms to block their disease transmission potential.

Although the genome showed low heterozygosity, the *C. brevitarsis* assembly was generated from pooled individuals due to practical constraints, including its small body size (1–3 mm), the lack of established laboratory colonies, and logistical challenges in sample collection from remote area and secure air transport to high containment facility, located approximately 1600 km away. Orthologs of genes previously associated with differential responses to BTV infection in *C. sonorensis* were identified, providing a comparative framework for future studies. However, *C. brevitarsis* remains a challenging species for experimental investigation due to the lack of established laboratory colonies, limiting direct functional validation. In this context, model systems such as *C. sonorensis*, and potentially species more closely related to *C. brevitarsis*, including the major arbovirus vector *C. imicola*, remain important for advancing understanding of vector–virus interactions. Future work will benefit from species-specific transcriptomic data and comparative analyses across *Culicoides* species to further explore genomic variation associated with vector biology.

This study presents the first chromosome-level genome assembly of an Australasian BTV vector species and identifies key gene classes with potential roles in regulating vectorial capacity. Compared with other biting midge taxa, the study demonstrated the highest BUSCO genome completeness, along with distinct repeat classes. Comparative genomic analyses across ten taxa revealed a clear clustering of the biting midges taxon from mosquitoes and sand flies, together with evidence of family- and species-specific gene family evolution, associated with vector-specific gene families. Furthermore, a curated set of vector-competence-associated genes and their genomic loci is proposed, offering a foundational resource for future studies aimed at understanding and mitigating *Culicoides*-transmitting viruses.

## Supplementary Information

Additional file 1: Figure S1. Identification and overlap of immune-related genes in *C. brevitarsis*. Venn diagram showing the overlap of immune-related genes identified from Gene Ontology and KEGG annotationsand orthology-based searches using reference immune gene sets from *D. melanogaster, Ae. aegypti, Ae. albopictus, Ar. subalbatus*, and *H. xiaojinensis*. Numbers in parentheses indicate the total number of genes identified from each source, while values within intersections represent shared genes. In total, 661 immune-related genes were identified in the *C. brevitarsis* genome.

Additional file 2: Table S1. A summary of annotation statistics of *C. brevitarsis*. Table S2. A table of immunity related genes, reported in *C. brevitarsis*. The table provides detailed gene information, including chromosome/gene/protein accessions, genomic coordinates, strand orientation, and presence across the different reference datasets used in this study. Table S3. Full list of vector competence–related genes associated with bluetongue virusin *C. brevitarsis* and their evolutionary conservation across Diptera. Genetic and biochemical details, and where they could be estimated, dN/dS estimates of all BTV-vector competence related genes in *C. brevitarsis* for which an ortholog was found in at least one of the other 11 dipteran taxa. Red cells indicate dN/dS > 1.00, grey indicate no significant deviation from neutrality, and green indicate dN/dS < 1.00. *C. brevitarsis* ortholog details are added on right.

## Data Availability

The paired-end Illumina and Nanopore sequencing raw data are available as part of NCBI BioProject PRJNA1059659. RNA sequences of two mRNA and three total RNA libraries can be found under accessions SRX23532275-9. All bioinformatic tools used are publicly available; the relevant articles corresponding to each tool have been cited appropriately. Genome annotations of *C. sonorensis* and *C. stellifer* are available at CSIRO data access portal (10.25919/1tf9-bn03). The codes are available at https:/github.com/asifratul/culicoides_brevitarsis_manuscript_codes
