# Supplementary figures and images for: Chromosome-scale genome of Culicoides brevitarsis provides a resource for comparative and vector biology studies
Source: Parasit Vectors. 2026 May 23;19:292. doi: 10.1186/s13071-026-07436-8 (PMC13374220; doi:10.1186/s13071-026-07436-8)

## Slide 1
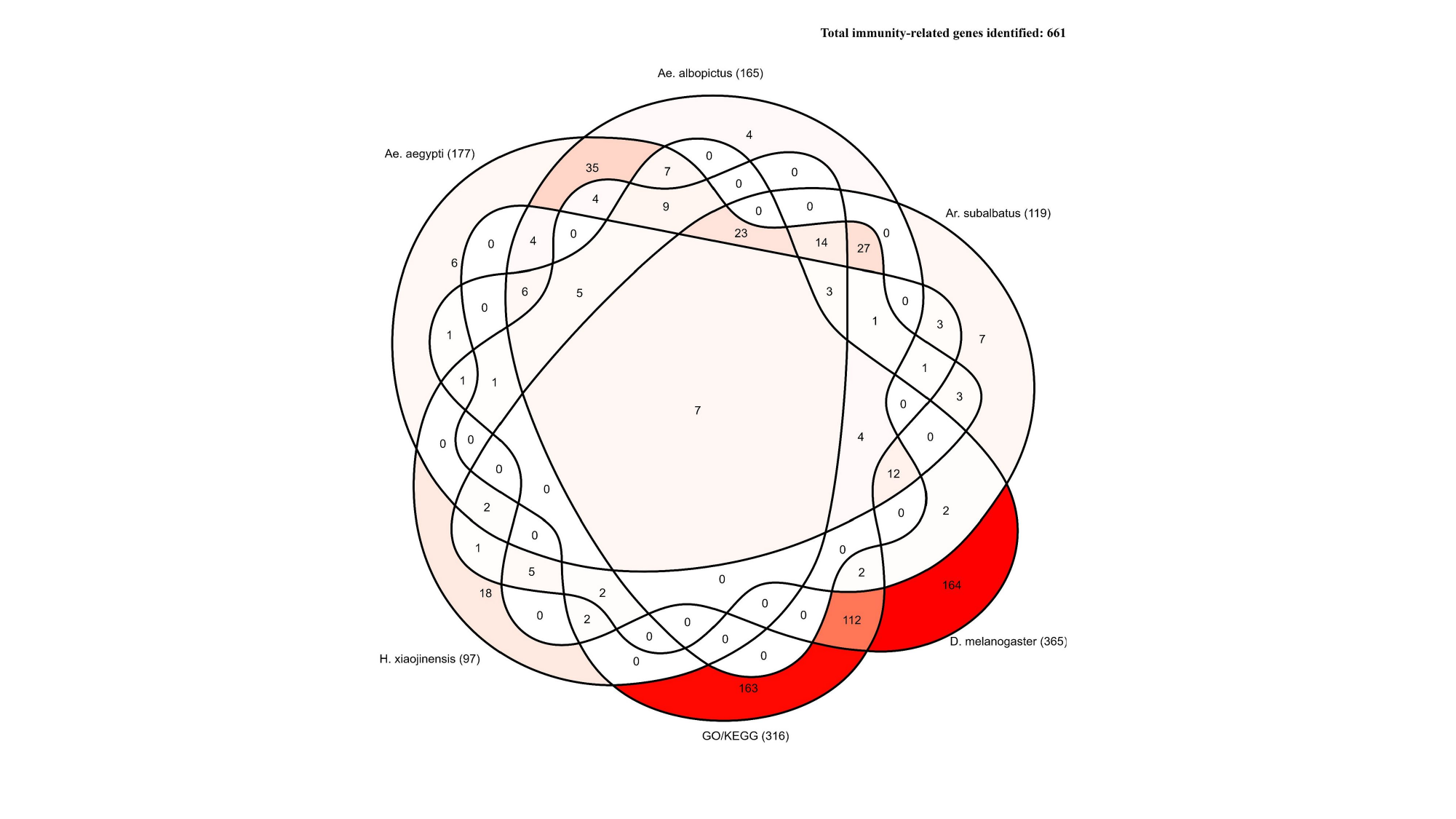

Supplement: Supplementary file 1 — Additional file 1: Figure S1. Identification and overlap of immune-related genes in C. brevitarsis. Venn diagram showing the overlap of immune-related genes identified from Gene Ontology and KEGG annotationsand orthology-based searches using reference immune gene sets from D. melanogaster, Ae. aegypti, Ae. albopictus, Ar. subalbatus, and H. xiaojinensis. Numbers in parentheses indicate the total number of genes identified from each source, while values within intersections represent shared genes. In total, 661 immune-related genes were identified in the C. brevitarsis genome. [file 13071_2026_7436_MOESM1_ESM.pptx]
